# Retraction Note: MicroRNA-15b shuttled by bone marrow mesenchymal stem cell-derived extracellular vesicles binds to WWP1 and promotes osteogenic differentiation

**DOI:** 10.1186/s13075-022-02950-3

**Published:** 2022-11-10

**Authors:** Yanhong Li, Jing Wang, Yanchao Ma, Wenjia Du, Haijun Feng, Kai Feng, Guangjie Li, Shuanke Wang

**Affiliations:** Department of Orthopaedics, Lanzhou University Second Hospital, No. 82, Cuiyingmen, Chengguan District, Lanzhou, 730030 Gansu Province People’s Republic of China

**Retraction Note: Arthritis Res Ther 22, 269 (2020)**

**https://doi.org/10.1186/s13075-020-02316-7**

The authors have retracted this article due to an overlap of images in figures 7B, figure 4H and Figure 5B. The authors no longer have confidence in the reliability of the results presented in this article. All authors agree to this retraction.

